# Relative validity of an online short FFQ assessing the Dutch adapted version of the Mediterranean-DASH Intervention for Neurodegenerative Delay (MIND) diet in older adults at risk of cognitive decline

**DOI:** 10.1017/S000711452510603X

**Published:** 2026-04-28

**Authors:** Sonja Beers, Sofie van Houdt, C. P. G. M. (Lisette) De Groot, Hanne B. T. de Jong, Kay Deckers, Lisa Waterink, Nynke Smidt, Joukje M. Oosterman, Sebastian Köhler, Marissa D. Zwan, Esther Aarts, Ondine van de Rest, Marian A. E. de van der Schueren

**Affiliations:** 1 Division of Human Nutrition and Health, Wageningen University & Research, Wageningen, The Netherlands; 2 Department of Nutrition, Dietetics and Lifestyle, HAN University of Applied Sciences, Nijmegen, The Netherlands; 3 Department of Psychiatry and Neuropsychology, Alzheimer Centrum Limburg, Mental Health and Neuroscience Research Institute (MHeNs), Maastricht University, PO Box 616, 6200 MD, Maastricht, The Netherlands; 4 Alzheimer Center Amsterdam, Neurology, Vrije Universiteit Amsterdam, Amsterdam UMC Location VUmc, Amsterdam, The Netherlands; 5 Amsterdam Neuroscience, Neurodegeneration, Amsterdam, The Netherlands; 6 Department of Epidemiology, University of Groningen, University Medical Center Groningen, Groningen, The Netherlands; 7 Radboud University, Donders Institute for Brain, Cognition and Behaviour, Nijmegen, The Netherlands

**Keywords:** Relative validity, Construct validity, Food record, Short FFQ, Older adults

## Abstract

A short FFQ was developed for online assessment of adherence to the Dutch Mediterranean-DASH Diet Intervention for Neurodegenerative Delay (MIND-NL) diet, a culturally adapted version of the original American MIND diet. This study aimed to evaluate the relative validity of this short FFQ for assessing adherence to the MIND-NL diet, as scored by the MIND-NL score, compared with 3-d food records among community-dwelling older adults at risk of cognitive decline (*n* 1078; 67·4 (sd 4·6) years; 64 % female). A combination of statistical methods was used to assess the relative validity: presence of bias by Bland–Altman analysis; strength of association with Kendall’s Tau-b and Spearman correlation coefficients and levels of agreement with Wilcoxon signed rank test, cross-classification and weighted Kappa (κ) statistics. The Kendall’s Tau-b correlation for the MIND-NL score was 0·33 (95 % CI: 0·29, 0·37; de-attenuated Tau-b: 0·45). Individual MIND-NL diet component score correlations ranged from 0·05 to 0·56, with 12 out of 15 of the MIND-NL diet components adequately correlated (> 0·20). The average MIND-NL scores for the short FFQ (8·4 (sd 1·8) points) and food records (6·7 (sd 1·7) points) showed to be significantly different (*P* < 0·001). The Kappa (κ) coefficient for tertile classification of the MIND-NL score was 0·29 (95 % CI: 0·25, 0·33), indicating an acceptable level of agreement in ranking participants beyond chance. Acceptable agreements (κ > 0·20) were observed for 10 out of 15 MIND-NL diet components. Taking all analyses together, the short FFQ showed acceptable validity for ranking older adults at risk of cognitive decline according to their adherence to the MIND-NL diet.

There is growing attention for dietary patterns that may contribute to slowing down cognitive decline and preventing dementia in later life^(1,2)^. The Mediterranean-DASH Diet Intervention for Neurodegenerative Delay (MIND) diet has specifically been developed with this aim^(3)^. The MIND diet is an originally American food-based dietary pattern and, thus, may not be optimal for populations with different dietary cultures and practices different from the American population. Therefore, culturally adapted versions are needed for use in these other populations^(4)^.

The MIND diet has already been adapted to a French, Chinese, Korean and Malaysian modified version^(5–8)^. In the Netherlands, the MIND diet has been adapted into the Dutch Mediterranean-DASH Diet Intervention for Neurodegenerative Delay (MIND-NL) diet, along with an accompanying MIND-NL diet scoring system. For the MIND-NL diet score, serving sizes were translated into quantities (weights in grams or volumes in millilitres) and adjusted to align with Dutch eating practices^(9)^. The MIND-NL score, ranging from 0 to 15 points, is considered to effectively capture the same dietary construct as the original American MIND diet, but needs evaluation.

Adherence to the MIND diet can be scored using 24-h recalls, food records or an FFQ. In the current literature, it is mostly assessed with FFQ, but most of these FFQ were not specifically designed to assess all MIND diet food groups^(10)^. Furthermore, the use of recalls, records and full-length FFQ can be relatively time-consuming and burdensome for older participants, especially if the sole interest is assessing adherence to the MIND diet.

Therefore, an online short Dutch FFQ, the ‘Eetscore-FFQ’, was adapted to assess the food groups specific to the MIND-NL diet^(11)^. This MIND-NL-Eetscore-FFQ was developed for use in research and clinical settings to easily assess adherence to the MIND-NL diet and to monitor dietary changes. While the Eetscore-FFQ has proven to be acceptable in ranking participants according to their dietary adherence to the Dutch Dietary guidelines of 2015, the MIND-NL adherence assessed by the MIND-NL-Eetscore-FFQ still requires evaluation. Additionally, although the Eetscore-FFQ has been validated in a population with a broad age range (19–91 years), it has not specifically been evaluated in a population at risk of cognitive decline^(11)^. Given the increasing focus on recruiting at-risk participants in dementia prevention studies, it is important to validate the short MIND-NL-Eetscore-FFQ within this population of interest.

In the present study, we therefore aimed to evaluate the relative validity of the short online MIND-NL-Eetscore-FFQ to assess adherence to the MIND-NL diet, using 3-d food records as a reference, in older adults at risk of cognitive decline. Additionally, as part of construct validation, we aimed to examine whether adherence to the MIND-NL diet accurately reflects the intake of nutrients known to support brain health.

## Methods

### Participants

For the current study, we used baseline data of the FINGER-NL study, a multicentre, multidomain lifestyle-intervention randomised, controlled trial conducted in the Netherlands aimed at maintaining of optimal cognitive function of older adults at-risk for cognitive decline (ID: NCT05256199). Details of the study have been described elsewhere^(12)^. Briefly, participants of the total FINGER-NL study ranged in age from 60 to 80 years of age, with 64 % being female. Participants had either subjective cognitive complaints or first-degree family with dementia and had at least two modifiable risk factors for dementia. Participants with dementia or mild cognitive impairment (self-reported) were excluded. The full list of in- and exclusion criteria can be found in online Supplementary Table S1. The FINGER-NL study was approved by the Medical Ethical Committee VU Medical Centre (NL77242.029.21; Amsterdam, The Netherlands) and conducted according to the Declaration of Helsinki. All participants provided written consent.

The total FINGER-NL study included 1210 older adults from the Netherlands. For the current evaluation of the MIND-NL-Eetscore-FFQ, only participants with completed baseline data of the MIND-NL-Eetscore-FFQ and one or more food records were included. Furthermore, plausible under- and over-reporters of the food records were excluded based on the following criteria: average daily energy intake of men outside the range of 800–4200 kcal/d and for women outside the range of 500–3500 kcal/d^(13)^.

All participants with at least one food record were included, as consistent results were observed between participants with one *v*. three food records.

### Study design

This is a cross-sectional study of baseline (i.e., pre-randomisation) data of the FINGER-NL trial. Data were collected between February 2022 and July 2023. For each participant, the MIND-NL-Eetscore-FFQ and the food records were distributed within the same week.

### MIND-NL-Eetscore-FFQ

The development of the MIND-NL-Eetscore-FFQ has been described previously^(9)^. This short FFQ is based on the Eetscore-FFQ, which showed acceptable ranking ability based on Bland–Altman plot and a Kendall’s tau-b correlation of 0·51 (95 % CI: 0·47, 0·55)^(11)^. The MIND-NL-Eetscore-FFQ includes seventy-two food items and forty-one questions and assesses both the Dutch Healthy Diet index (DHD2015-index) and the MIND-NL score^(14)^. For the MIND-NL score, only forty-six food items and twenty-seven questions are relevant. The questions are chronologically ordered, from breakfast to late evening. The reference period is the previous month. Intakes are estimated using standard portion sizes and commonly used household measures. Average daily intake, in grams, of food items is calculated by multiplying frequency of consumption by portion size. The MIND-NL-Eetscore-FFQ is self-administered using a web-based assessment tool (https://data.eetscore.nl ).

### Three-Day food records

Food records were used as the reference method and were administered through a smartphone application (Traqq®)^(15)^. Three days were randomly assigned to the participant, i.e. two weekdays and one weekend day. Participants received a notification at 08.00 each morning to track their food intake for the day using an extensive food list based on the Dutch Food Composition Database (NEVO 2016)^(16)^. Food products could be reported as household measures, standard portion sizes or as amounts in grams. Products participants could not find in the food list or forgot to enter could be recorded on paper. Products recorded on paper were later coded by trained researchers as the best-fitting product from the Food Composition Database.

Data of the food records were entered in the computation module of Compl-eat™^(17)^. Energy and nutrient intakes were calculated using the 2016 Dutch Food Composition Database. Data were reviewed by a trained dietician, with particular focus on reported amounts, in order to identify and correct unusual amounts using standardised portion sizes.

### MIND-NL Score

The MIND-NL score comprises fifteen MIND diet components (Table 1). The scoring for MIND-NL is based on quantities. The development of the MIND-NL scoring is described elsewhere,^(9)^ and the scoring is summarised in Table 1. For each component, a score of 0, 0·5 or 1·0 can be assigned. A score of 1·0 indicates full adherence to the MIND-NL diet component. Summing all component scores yields a total MIND-NL score ranging from 0 to 15 points.

**Table 1.** Components of the Dutch versions of the Mediterranean-DASH diet intervention for Neurodegenerative Delay (MIND-NL) diet and their cut-off values

Components	Unit	Score		
		Minimum 0·0	Moderate 0·5	Maximum 1·0
1. Green leafy vegetables	(g/d)	≤29	>29 − <100	≥100
2. Other vegetables	(g/d)	<71	≥71 − <100	≥100
3. Berries and strawberries	(g/d)	<7	≥7 − <36	≥36
4. Whole grains	(g/d)	<30	≥30 − <90	≥90
5. Nuts	(g/d)	<3	≥3 − <20	≥20
6. Fish – not fried	(g/d)	<13	13	>13
7. Poultry – not fried and skinless	(g/d)	<14	≥14 − <29	≥29
8. Beans and legumes	(g/d)	<9	≥9 − <26	≥26
9. Olive oil	(g/d)	<15	≥15 − <30	≥30
10. Full-fat cheese	(g/d)	≥30	>9 − <30	≤9
11. Butter and sticky margarines	(g/d)	≥10	>5 − <10	≤5
12. Red and processed meat	(g/d)	≥100	>43 − <100	≤43
13. Take out, fried foods and snacks	(serving eq./week)	>3	>1 − ≤3	≤1
14. Cookies, pastries and sweets	(serving eq./week)	>4	>2 − ≤4	≤2
15. Wine	(ml/d)	>100	NA	≤100

Cut-off values based on the article of Beers S & van Houdt S et al. (2024)^(9)^. The cut-off values were transformed to daily intakes, except take out foods, fried foods and snacks and cookies, pastries and sweets.

#### Three-Day food records

For the assessment of the MIND-NL score from the food records, the Dutch Food Composition Database, in particular the assigned product (NEVO) code, was used. A summary of the assigned products to specific MIND-NL diet components can be found in online Supplementary Table S2. For each MIND-NL diet component, total food intake was calculated and averaged to determine daily intake. Component scores were then derived from these average daily intakes.

### Participant characteristics

General participant characteristics (i.e. age, educational level, smoking status and subjective memory complaints) were acquired via single questions. Participants were screened for their cognitive status using the Montreal Cognitive Assessment^(18)^. Height was measured without shoes using a stadiometer (SECA 213; SECO Corp.), and weight was assessed without shoes and heavy clothing. BMI was calculated as weight/height^2^. Further baseline characteristics can be found in the design paper of FINGER-NL^(12)^.

### Statistical analysis

General characteristics are presented as means and sd or median and interquartile range. Differences in characteristics between men and women were tested using an independent *t* test or Mann–Whitney *U* test for continuous data, depending on the normality of the distribution. Categorical data were tested with a *χ* ^2^ test. The Mann–Whitney *U* test was used for MIND-NL diet scores due to its ordinal origin. Because of the significant differences in MIND-NL diet scoring between men and women, further analysis was done with sex-specific MIND-NL diet tertiles.

To assess the relative validity between the MIND-NL-Eetscore-FFQ and food records, a combination of statistical methods was applied^(19)^. The presence of bias was assessed by the Bland–Altman analysis, strength of association with Kendall’s Tau-b and Spearman correlation coefficients and levels of agreement with Wilcoxon signed rank test, cross-classification and weighted Kappa (κ) statistics. Additionally, these analyses were conducted in subgroups of participants with and without subjective memory complaints to determine whether the validity of the FFQ differs between these groups, given that memory complaints might affect accurate dietary recall.

In the Bland–Altman analysis, average MIND-NL scores from the two dietary assessment methods for total MIND-NL score were plotted against the mean differences. Systematic difference between the two dietary assessment methods (systemic bias) and the extent to which the two methods agree (limits of agreement) were evaluated. Proportional bias was assessed by fitting a regression line to the plot. This line examines the association between the average MIND-NL scores and the mean differences. A significant slope (*P* ≤ 0·05) indicates that discrepancies between the two dietary assessment methods vary according to their average MIND-NL score.

Kendall’s Tau-b (τb) correlation coefficients, which accounts for tied ranks in the data, were determined. In addition, Spearman’s rank (ρ) correlation coefficients were used to enable comparison with other studies. CI for the correlation coefficients were calculated using Fisher’s Z-transformations. Kendall’s tau-b (τ_b_) correlations and Spearman’s Rank (*ρ*) correlation were additionally adjusted for day-to-day variability according to the method as described by Rosner and Willet, multiplying the crude correlation coefficients with a factor sqrt (1 + (σintra2/σinter2)/n), where n is the number of repeated food records per participant, σintra2 is the intra-individual variance and σinter2 is the inter-individual variance between the food records^(20)^. Correlation coefficients were both determined for the total MIND-NL score, as well as for the individual MIND-NL diet components. Correlation coefficients of < 0·20 were considered to be poor, 0·20–0·49 acceptable and ≥ 0·50 good^(19)^.

The Wilcoxon signed rank test assessed the statistical difference between the average MIND-NL scores of the two dietary assessment methods. Cross-classification evaluated the proportion of participants assigned to the same or opposite MIND-NL diet score tertile. The weighted Kappa (κ) coefficients were calculated to assess the level of agreement beyond chance between the MIND-NL tertile scores and the individual MIND-NL diet component scores from the two methods. Kappa (κ) coefficients between 0·20 and 0·60 indicate an acceptable level of agreement and a κ ≥ 0·60 a good level of agreement^(19)^.

Additional analyses were conducted to assess construct validity by examining trends in average daily macro- and micronutrient intake across levels of MIND diet adherence. The MIND diet focuses on specific nutrients that were considered by the developers of the MIND diet to support brain health and reduce neurodegenerative diseases. Nutrients that are emphasised in the MIND diet are B vitamins (B_12_, folate, B_6_ and niacin), vitamin E, carotenoids (especially beta-carotene and lutein), flavonoids and other polyphenols and *n*-3 fatty acids. Conversely, the diet recommends limiting the intake of saturated fats^(3,21–23)^. Nutrient intake trends were analysed across sex-specific tertiles of the MIND-NL score using either ANOVA or the Kruskal–Wallis test, depending on the variable’s distribution. Macronutrient intake is reported both as actual intake (g/d) and as a percentage of total energy intake (E%). Micronutrient intake is reported as energy-adjusted intake, standardised to intake per 2000 kcal.

All data were analysed using RStudio Version 1.4.1717.

## Results

### Participant characteristics

Of the 1210 participants, a total of 1136 participants completed the MIND-NL-Eetscore-FFQ, and 1174 participants had at least data of 1-d food records. A total of thirty-one participants were excluded due to implausible daily average energy intake, of which thirty were identified as under-reporters. Therefore, the final study sample consisted of 1078 participants with complete and reliable dietary intake data for the analysis of the relative validity of the MIND-NL Eetscore-FFQ.

Participant characteristics of the 1078 participants are presented in Table 2. The mean age of the total study population was 67·4 (sd 4·6) years; 64 % of the participants were female, and mean BMI was 28·2 (sd 4·2) kg/m^2^. The average Montreal Cognitive Assessment score was 26·7 (sd 2·1), whereby 72·9 % of the participants reported subjective memory complaints. Men had a significantly lower mean Montreal Cognitive Assessment score (*P* < 0·001). Women were significantly younger (*P* < 0·001) on average and had a lower BMI (*P* = 0·02) compared with men.

**Table 2.** General characteristics, MIND-NL score and its absolute intake of MIND-NL components for the total population and for men (N=387) and women (N=691) separated, based on the food records

	Total (*n* 1078)				Men (*n* 387)				Women (*n* 691)				P^†^
	Mean^*^	SD^*^	n	%	Mean^*^	SD^*^	n	%	Mean^*^	SD^*^	n	%	
Age (years)	67.4	4.6			68.1	4.6			67.0	4.5			**<0.001**
BMI (kg/m^2^)	28.2	4.2			28.6	3.8			28.0	4.4			**0.02**
Education level^‡^													
Low			141	13.1			45	11.6			96	13.9	0.28
Middle			264	24.5							176	25.5	
High			672	62.4			254	22.7 65.6			418	60.6	
Smoking status													
Current			34	4.0			17	4.4			26	3.7	**0.02**
Former			675	62.6			263	68.0			412	59.6	
Never			360	33.4			107	27.6			253	36.6	
MOCA score^§^	26.7	2.1			26.0	2.2			27.1	2.0			**<0.001**
Subjective memory complaints			785	72.9			298	72.7			512	73.4	0.93
Energy intake (kcal)	1643.9	481.5			1817.0	510.0			1547.0	481.5			**<0.001**
MIND score	6.7	1.7			6.2	1.6			6.9	1.7			**<0.001**
1. Green leafy vegetables (g/d), median [IQR]	0.0	25.0			0.0	20.0			4.7	26.3			**0.004**
2. Other vegetables (g/d), median [IQR]	140.0	155.0			120.2	119.9			155.1	165.9			**<0.001**
3. Berries and strawberries (g/d), median [IQR]	0.0	33.3			0.0	16.8			0.0	33.3			**<0.001**
4. Whole grains (g/d), median [IQR]	80.0	73.3			93.3	92.5			75.0	61.5			**<0.001**
5. Nuts (g/d), median [IQR]	8.3	20.6			5.0	19.8			8.3	21.7			**0.01**
6. Fish (g/d), median [IQR]	0.0	28.3			0.0	2.50			0.0	31.2			0.27
7. Poultry (g/d), median [IQR]	0.0	0.0			0.0	0.0			0.0	2.5			**0.03**
8. Beans and legumes (g/d), median [IQR]	0.0	0.0			0.0	0.0			0.0	1.9			**0.004**
9. Olive oil (g/d), median [IQR]	0.0	2.2			0.0	0.0			0.0	3.3			**<0.001**
10. Full-fat cheese (g/d), median [IQR]	13.3	26.7			13.3	30.0			13.3	26.7			0.78
11. Butter and sticky margarines (g/d), median [IQR]	1.6	5.4			0.6	3.9			1.9	6.0			**<0.001**
12. Red and processed meat (g/d), median [IQR]	46.3	62.8			62.3	70.3			36.7	54.3			**<0.001**
13. Take out, fried foods, and snacks (eq./wk), median [IQR]	0.7	3.2			1.5	4.2			0.3	2.6			**<0.001**
14. Cookies, pastries, and sweets (eq./wk), median [IQR]	4.6	5.6			5.0	5.4			4.2	5.6			**0.03**
15. Wine (ml/d), median [IQR]	0.0	83.3			0.0	100.0			0.0	73.3			0.38

Abbreviations: BMI, Body Mass Index; MOCA, Montreal Cognitive Assessment.

All *P*-values in boldface are significant based on <0.05 cut-off.

* Presented as mean and SD, otherwise stated.

† Normally distributed values tested with ANOVA, non-normally distributed data tested with Kruskal Wallis test.

‡ Education level was categorized into low, medium and high based on the International Standard Classification of Education (ISCED 2011) guideline.

§ A theorical range between 0-30, a score of ≥26 indicates normal cognition.

### Three-Day food records and MIND-NL score

Of all participants, 90·7 % completed 3 d of food records, 7·1 % completed 2 d and 2·1 % completed only 1 d. The mean MIND-NL score for the total population, based on the food record data, was 6·7 (sd 1·7) (range 1·5–12·0). The MIND-NL score was significantly higher in women compared with men, while energy intake was significantly lower in women compared with men (Table 2).

### Relative validity of MIND-NL-Eetscore-FFQ

**Figure 1.**
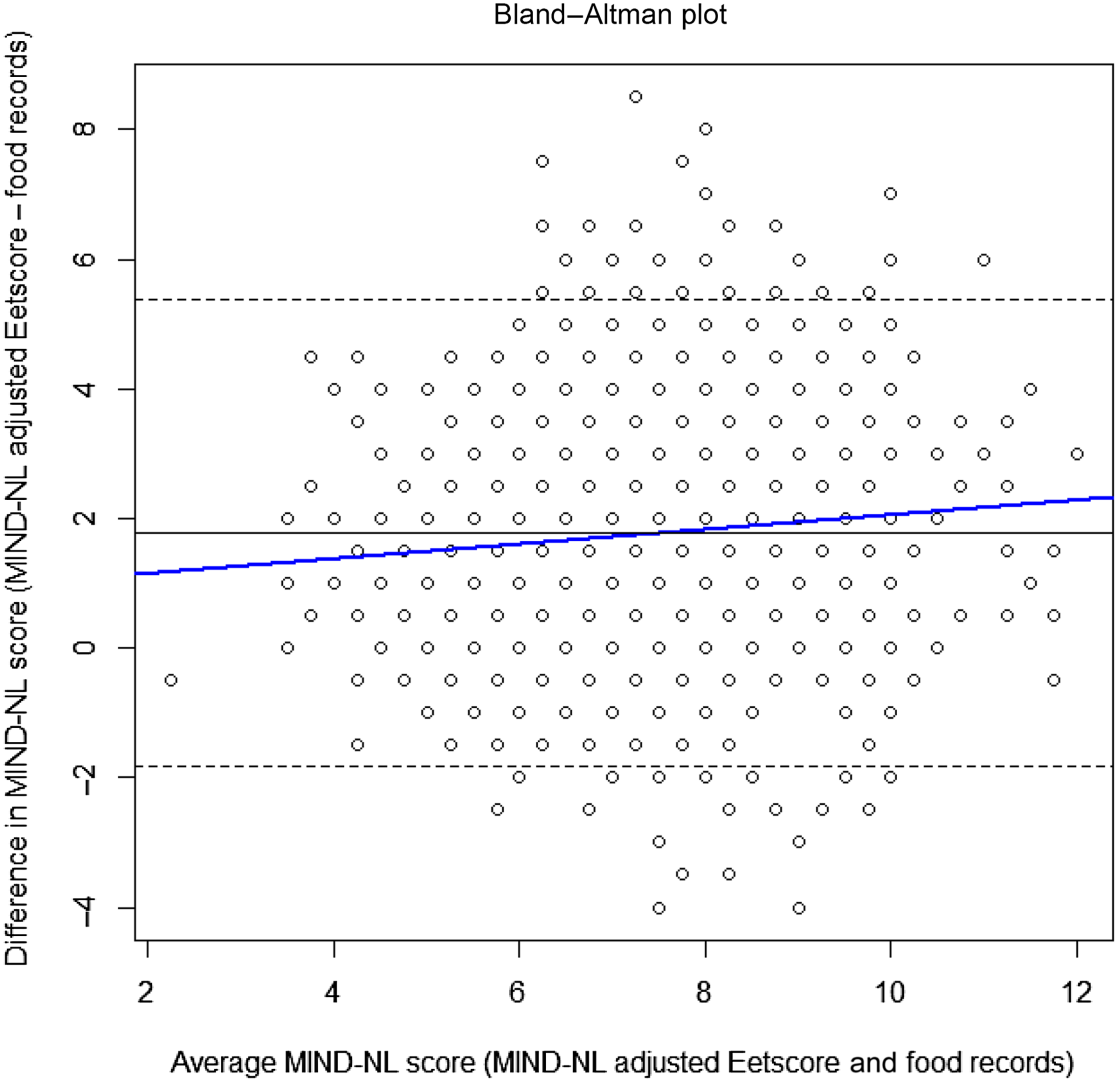
Bland–Altman plot of the differences in scoring of the MIND-NL diet with the food record and the MIND-NL-Eetscore-FFQ, plotted against mean of both methods. Mean difference (black solid line), 95 % limits of agreement (1·96 × sd of mean difference; black dashed line) and linear regression line (blue solid line) are included. MIND-NL, Dutch versions of the Mediterranean-DASH diet intervention for Neurodegenerative Delay.

The mean MIND-NL score, based on the MIND-NL-Eetscore-FFQ, was 8·4 (sd 1·8) points (range 2·0–14·0 points), which was significantly higher compared with the mean score derived from the food records (mean difference = 1·7 (sd 1·8) points, *P* < 0·001). There was a relative wide limits of agreement (–1·8 and 5·4 points). The regression line within the Bland–Altman plot was significant (*β* = 0·11, *P* = 0·003); the higher the average MIND-NL score, the higher the discrepancy between the two methods (Figure 1). The mean difference of the MIND-NL score between the two assessment methods did not differ between men (1·8 (sd 1·8)) and women (1·8 (sd 1·9)).

The Kendall’s tau-b (τ_b_) correlation coefficient between the two methods for the total MIND-NL score was 0·33 (95 % CI: 0·29, 0·37), indicating an acceptable strength of correlation (Table 3). The correlation between the individual MIND-NL component scores ranged between 0·05 and 0·56. The lowest correlations (< 0·20) were observed for the components olive oil, green leafy vegetables and legumes. All other components were acceptable, with the highest correlations for the components wine (0·55), full-fat cheese (0·39) and whole grains (0·38) (Table 3). After correcting for day-to-day variation in the food records, olive oil was also assessed to have a just acceptable strength of correlation (de-attenuated τ_b_ = 0·20) (Table 3).

**Table 3.** Adherence to the MIND-NL diet and its components, Kappa (κ) statistics, Kendall’s Tau-b correlation coefficients and spearman correlation coefficients between the MIND-NL-Eetscore-FFQ and the food records (*n* 1078)

	Food records^*^		MIND-NL-Eetscore-FFQ^*^									
	Mean	sd/%	Mean	sd/%	Kappa (κ)	95 % CI	τ_b_	95 % CI	De-attenuated τ_b_^†^	ρ	95 % CI	De-attenuated ρ^†^
MIND-NL score, mean (sd)	6·7	1·7	8·4	1·8	0·29	0·25, 0·33	0·33	0·29, 0·37	0·45	0·44	0·39, 0·49	0·49
1. Green leafy vegetables		3·0		27·6	0·05	0·03, 0·07	0·13	0·09, 0·17	0·19	0·14	0·08, 0·20	0·21
2. Other vegetables		66·9		30·1	0·16	0·12, 0·20	0·23	0·19, 0·26	0·28	0·24	0·19, 0·30	0·32
3. Berries and strawberries		18·0		17·0	0·30	0·23, 0·33	0·34	0·30, 0·38	0·41	0·37	0·32, 0·42	0·45
4. Whole grains		43·2		72·1	0·27	0·23, 0·31	0·38	0·34, 0·41	0·41	0·40	0·35, 0·45	0·47
5. Nuts		26·6		36·1	0·28	0·23, 0·33	0·35	0·31, 0·38	0·41	0·39	0·33, 0·44	0·48
6. Fish		32·5		65·2	0·23	0·18, 0·27	0·28	0·24, 0·32	0·36	0·28	0·23, 0·34	0·38
7. Poultry		17·3		37·7	0·16	0·12, 0·19	0·22	0·18, 0·26	0·28	0·23	0·18, 0·29	0·30
8. Beans and legumes		11·6		20·6	0·06	0·02, 0·10	0·10	0·06, 0·14	0·13	0·11	0·05, 0·16	0·14
9. Olive oil		0·6		12·2	0·02	−0·00, 0·05	0·05	0·01, 0·09	0·20	0·05	−0·01, 0·11	0·20
10. Full-fat cheese		42·9		43·8	0·35	0·30, 0·39	0·39	0·35, 0·42	0·46	0·42	0·37, 0·47	0·54
11. Butter and sticky margarines		74·5		55·6	0·23	0·18, 0·27	0·27	0·23, 0·30	0·28	0·28	0·23, 0·34	0·32
12. Red and processed meat		47·2		55·2	0·27	0·22, 0·31	0·31	0·28, 0·35	0·39	0·34	0·28, 0·39	0·45
13. Take out, fried foods and snacks		53·3		43·3	0·21	0·16, 0·26	0·24	0·20, 0·28	0·33	0·26	0·21, 0·32	0·38
14. Cookies, pastries and sweets		24·2		32·7	0·32	0·27, 0·36	0·36	0·32, 0·39	0·44	0·40	0·35, 0·45	0·53
15. Wine		75·8		77·6	0·56	0·50, 0·61	0·56	0·53, 0·58	0·58	0·56	0·51, 0·59	0·62

MIND-NL, Dutch Mediterranean-DASH Diet Intervention for Neurodegenerative Delay; MIND, Mediterranean-DASH Diet Intervention for Neurodegenerative Delay; τ_b,_ kendall’s Tau-b correlation coefficient; ρ, Spearman’s rank correlation coefficient.

* Proportion of participants scored maximum score (1·0 = optimal adherence) for each MIND-NL component, except for MIND-NL score which is given as mean (sd).

† Corrected for day-to-day variation of the repeated food recalls (Rosner and Willet, 1988).

Based on the MIND-NL tertiles, results of cross-classification showed that 48·0 % of participants were assigned to the same tertile, and 10·1 % were classified in the opposite tertile. The Kappa (κ) coefficient calculated between the tertile scores of the MIND-NL diet was 0·29 (95 % CI: 0·25, 0·33), indicating an acceptable level of agreement in ranking beyond chance. For the individual components, ten out of fifteen components scored acceptable (κ ≥ 0·20) on the weighted Kappa (κ) statistics (Table 3).

#### Sub-analysis with and without subjective memory complaints

Differences in the analysis in the population without subjective memory complaints compared with the whole sample were seen for Bland–Altman analysis, correlation and agreement (Kappa) of the component full-fat cheese. The regression line within the Bland–Altman plot was not significant (*β* = –0·02, *P* = 0·75). The Kendall’s tau-b (τ_b_) correlation coefficient and Kappa (κ) coefficient of full-fat cheese were lower compared with the whole sample (τ_b_ = 0·28, 95 % CI: 0·21, 0·35, κ = 0·25, 95 % CI: 0·16, 0·34). No other differences were found between the sub-groups with and without subjective memory complaints compared with whole group analysis (online Supplementary Figure S1, Figure S2, Table S3, Table S4)

### Construct validity of MIND-NL diet

Based on MIND-NL diet tertiles derived from the MIND-NL-Eetscore-FFQ and nutrient data of the food records, energy intake did not significantly differ between the MIND-NL tertiles (*P* = 0·42) (Table 4). Of the macronutrients, fibres, *n*-3 fatty acids and total and plant-based proteins were positively associated with MIND-NL adherence, whereas saturated fat and alcohol were negatively associated with MIND-NL adherence. Of the energy-adjusted micronutrients, a significant positive trend was found between MIND diet adherence and all micronutrients, except for vitamin B_12_ (*P* = 0·09) and Na (*P* = 0·35) (Table 4). Using MIND-NL diet tertiles derived from the food records, vitamin B_12_ was positively associated with MIND-NL diet adherence, and the other significant trends of macro- and micronutrients were sustained (online Supplementary Table S5).

**Table 4.** Distribution of characteristics, macronutrients and selected micronutrient intakes across sex-specific tertiles of MIND-NL adherence. Tertiles derived from the MIND-NL-Eetscore-FFQ, macro- and micronutrient intake derived from the food records

	T1^*,†^		T2^*,†^		T3^*,†^		
	*n* 419		*n* 368		*n* 291		
	Mean/Median	sd/IQR	Mean/Median	sd/IQR	Mean/Median	sd/IQR	*P*_for trend_^‡^
MIND score, Range	6·7, *2·0–8·0*	1·0	8·7, *7·5–9·5*	0·6	10·7, *9·5–14·0*	0·9	**<0·001**
Age (years)	67·3	4·8	67·8	4·6	67·2	4·3	0·22
BMI	28·4	4·3	28·1	4·1	28·2	4·2	0·63
MoCA score	26·8	2·1	26·5	2·1	26·7	2·1	0·15
Daily macronutrient intakes							
Energy (kcal)	1659	493	1652	462	1612	488	0·42
Carbohydrates (g)	167	56	167	53	157	58	**0·03**
Carbohydrates (E%)	41·5	8·0	41·6	7·7	40·1	8·8	**0·03**
Mono- and disaccharides (g)	71	30	71	28	68	27	0·35
Fibre (g)	17	6	19	7	21	8	**<0·001**
Total fat (g)	68	27	66	25	67	26	0·71
Total fat (E%)	35·1	7·3	34·5	6·8	35·8	9·0	0·10
Saturated fat (g)	26	11	24	10	23	10	**<0·001**
*n*-3 fatty-acids (g), median (IQR)	1	1	2	1	2	1	**0·002**
Protein (g)	67	21	69	21	72	23	**0·010**
Protein (E%)	16·8	3·7	17·4	3·8	18·5	4·2	**<0·001**
Plant protein (g)	27	10	28	10	29	11	**<0·001**
Animal protein (g)	40	17	41	17	42	19	0·34
Alcohol (g), median (IQR)	5	15	5	14	1	9	**<0·001**
Alcohol (E%), median (IQR)	1·9	6·7	1·9	5·3	0·4	3·8	**<0·001**
Daily energy-adjusted micronutrient intakes							
Vitamin A RE/2000 kcal	665	589	871	792	904	719	**<0·001**
Beta-carotene ug/2000 kcal, median (IQR)	1210	2221	1925	3239	2232	3283	**<0·001**
Thiamin mg/2000 kcal	1	0·2	1	0·3	1	0·3	**<0·001**
Niacin mg/2000 kcal	17	7	18	5	19	6	**<0·001**
Vitamin B_6_ mg/2000 kcal	1	0·5	2	0·4	2	0·4	**<0·001**
Folate ug/2000 kcal	246	74	278	96	323	146	**<0·001**
Vitamin B_12_ ug/2000 kcal	5	5	5	9	6	8	0·09
Vitamin C mg/2000 kcal, median (IQR)	80	78	93	80	108	105	**<0·001**
Vitamin D ug/2000 kcal, median (IQR)	2	2	3	3	3	2	**0·003**
Vitamin E mg/2000 kcal, median (IQR)	11	6	11	5	13	6	**<0·001**
Vitamin K ug/2000 kcal, median (IQR)	79	104	102	116	141	182	**<0·001**
Mg mg/2000 kcal	342	70	373	77	409	103	**<0·001**
Fe mg/2000 kcal	11	2	12	3	12	3	**<0·001**
Ca mg/2000 kcal	984	349	993	339	1055	344	**0·02**
Na mg/2000 kcal	2363	730	2406	729	2323	747	0·35
Potassium mg/2000 kcal	3274	708	3500	781	3770	881	**<0·001**
Zn mg/2000 kcal	10	3	11	3	11	3	**<0·001**
Se/2000 kcal, median (IQR)	44	21	51	25·2	56	29	**<0·001**

MIND-NL, Dutch Mediterranean-DASH Diet Intervention for Neurodegenerative Delay; MOCA, Montreal Cognitive Assessment; IQR, interquartile range.

All *P*-values in boldface are significant based on <0.05 cut-off.

* Standard reporting mean (sd), otherwise stated.

† MIND-NL tertiles are sex-adjusted; *tertiles men* T1:2·0–7·0, T2:7·5–9·0. T3:9·5–14·0, *tertiles women* T1:2·0–8·0, 8·5–9·0, 9·5–14·0.

‡ Normal distributed data tested with ANOVA, non-normally distributed data tested with Kruskal–Wallis rank sum test for testing differences between MIND diet tertiles.

## Discussion

Our evaluation of the relative validity of the MIND-NL-Eetscore FFQ, compared with 3-d food records, suggests that the MIND-NL-Eetscore FFQ is acceptable for ranking participants by their adherence to the MIND-NL diet. A systematic difference was observed between the two methods, with the MIND-NL-Eetscore-FFQ yielding a higher average MIND-NL diet score than the food records. In assessing construct validity, higher adherence to the MIND-NL diet was positively associated with intakes of fibre, *n*-3 fatty acids and various micronutrients and negatively associated with intakes of saturated fats and alcohol.

The discrepancy between the MIND-NL scores based on the MIND-NL-Eetscore-FFQ and the 3-d food records can likely be attributed to the timeframe of recall. It is more likely for individuals to have consumed MIND-specific components such as legumes and green leafy vegetables over the past month than over three randomly assigned days. It is known that these MIND components are not eaten on a daily basis^(24)^. Indeed, these components showed the lowest agreement between the two methods. Olive oil also exhibited low agreement, which might be due to differences in how the two methods inquire about cooking oils; the brief FFQ explicitly asked about the use of cooking oils, whereas the food records did not. Consequently, the intake of cooking oils might not have been reported in food records, especially among those who do not cook their own meals.

Our results can also be explained by the expected types of measurement errors associated with these methods. FFQ tend to overestimate healthy food items compared with food records, primarily due to recall bias^(25)^. Our study shows this overestimation for the components whole grains, nuts and fish. This might also have contributed to the relatively higher MIND-NL scores observed in the MIND-NL-Eetscore-FFQ. Conversely, food records are more prone to random measurement error due to within-person day-to-day variation. After correcting for this variation, the Kendall’s tau-b correlation for olive oil improved to be acceptable compared with the non-corrected Kendall’s tau-b correlation. Despite including older adults with a high proportion of subjective memory complaints, which could potentially have affected the validity of the MIND-NL-Eetscore-FFQ due to memory bias, the MIND-NL score from the brief FFQ still correlated and ranked participants acceptably compared with the score derived from food records. Furthermore, subgroup analysis of participants without subjective memory complaints did not enhance the relative validity of the MIND-NL-Eetscore-FFQ.

A study, which evaluated an online screener of the Mediterranean Eating Pattern for American-III (MEPA-III) against a full-length FFQ in older adults with Parkinson’s disease, showed a Kappa coefficient (κ = 0·25) and Pearson’s correlation (*r* = 0·50) that were comparable to our study^(26)^. The MEPA-III shares many food components with the MIND-NL diet, including green leafy vegetables and berries. It should, however, be noted that the MEPA-III was validated against a different dietary assessment tool than the one we used in our study. Since both the MEPA-III screener and the reference FFQ introduced correlated errors due to the recall nature of the questionnaires, the validation of MEPA-III might be overestimated^(13)^. This indicates that the MIND-NL-Eetscore-FFQ is comparable, but without the possible overestimation due to correlated errors, in strength to another short FFQ assessing a dietary score with similar food components.

Although the Bland–Altman plot indicated the presence of proportional bias with a significant trend line, the clinical relevance of this finding is debatable. With each point increase in the average MIND-NL diet score, the difference between the two assessment methods increased by 0·11 points. Given that the MIND-NL increments are based on 0·5-point steps, a difference of 0·11 is unlikely to be meaningful in practical terms. Therefore, while the proportional bias is statistically significant, it is not likely to impact the overall interpretation of MIND-NL diet adherence when using the FFQ for ranking individuals.

The MIND-NL diet was developed as a Dutch adaptation of the MIND diet. Our study showed, with the assessment of construct validity, that the MIND-NL diet adequately reflects the original concept of the MIND diet, emphasising nutrients favourable for the brain, including B-vitamins, vitamin E, beta-carotene, anti-oxidants,and *n*-3 fatty acids, while deemphasising nutrients unfavourable for the brain, like saturated fatty acids^(21–23)^. Unfortunately, data on polyphenol content were not available within the Dutch Food Composition Database. However, we observed a positive trend across tertiles in the intake of berries and strawberries, which are known to be high in the polyphenol anthocyanin (online Supplementary Tables S6 and S7)^(27)^. The trends of nutrient intakes across MIND-NL tertiles are comparable with a French study that did not adapt the MIND diet score, except for energy and vitamin B_12_ intake^(28)^. In our study, energy intake was similar across MIND diet tertiles, while the French study showed a significant increase in energy intake with higher MIND diet tertiles. For vitamin B_12_, the results were opposite; our study found a positive trend of vitamin B_12_ intake across tertiles, whereas the French study did not observe this trend. Vitamin B_12_ is a crucial nutrient involved in multiple pathways important for brain health^(29)^. While plant-based diets often lack sufficient vitamin B_12_, the inclusion of fish and poultry in the MIND diet could have increased vitamin B_12_ intake^(30)^. Additionally, although dairy is not specifically captured in the MIND-NL diet, it can still contribute to overall B_12_ levels. Based on the trends of nutrient intakes across MIND-NL diet adherence, we can conclude that the MIND-NL diet score is valid in measuring the intended construct of the MIND diet.

Our study has several strengths. Among the self-reported comparison methods for the MIND-NL-Eetscore-FFQ, the food records can be considered the most appropriate. FFQ and food records likely have the least correlated errors, as food records do not rely on memory and are open-ended, unlike FFQ^(13)^. For evaluation validity, it is important to have a reference method with errors independent from the FFQ to avoid overestimating validity. Another strength is that both the food records and the MIND-NL-Eetscore-FFQ were assessed via electronic devices (mobile application and website, respectively), which reduces the workload for researchers and is believed to reduce bias by socially desirable responses of participants^(25)^. An online questionnaire can feel more anonymously and therefore can reduce the problem of socially considered answers. Additionally, the sample size was adequate, and the validation was conducted on a targeted study population relevant to research involving the MIND diet.

Limitations of this study can be found in its study design. The FINGER-NL study is a randomised controlled trial and therefore not specifically designed to assess the relative validity of the MIND-NL-Eetscore-FFQ. The FFQ and food records were sent out in the same week, while the brief FFQ queries retrospectively (past month). When the FFQ queries food intake before the period covered by food records, this might result in lower correlation coefficients^(13)^. While we assume that the FFQ measures habitual intake, the timing of the brief FFQ could have influenced the strength of agreement, possibly underestimating the correlations between the two dietary assessment methods. Another limitation of the study design is that we could not test for reproducibility. The original Eetscore-FFQ showed good to excellent reproducibility (ICC = 0·91). Given that the other aspects of validity for the MIND-NL-Eetscore-FFQ align with the validation of the original Eetscore-FFQ and that the questionnaire’s structure remains unchanged, we assume that its reproducibility will also be comparable to that of the original Eetscore-FFQ^(11)^.

To conclude, the MIND-NL-Eetscore-FFQ can be used to rank older adults at risk of cognitive decline according to their adherence to the MIND-NL diet. The MIND-NL diet adherence also distinguishes the nutrients important for brain health, herewith measuring the same construct as the original MIND diet. The MIND-NL-Eetscore-FFQ is valid for use in future studies assessing MIND-NL diet adherence in relation to neurodegenerative diseases.

## Supporting information

Beers et al. supplementary material 1 Beers et al. supplementary material

Beers et al. supplementary material 2 Beers et al. supplementary material

## Supplementary material

For supplementary material/s referred to in this article, please visit https://doi.org/10.1017/S000711452510603X
