# Supplementary material for: Relative validity of an online short FFQ assessing the Dutch adapted version of the Mediterranean-DASH Intervention for Neurodegenerative Delay (MIND) diet in older adults at risk of cognitive decline
Source: Br J Nutr. 2026 Jan 2;135(8):895–904. doi: 10.1017/S000711452510603X (PMC13315530; doi:10.1017/S000711452510603X)
Supplement: Beers et al. supplementary material 1 — Beers et al. supplementary material [file S000711452510603Xsup001.pdf]

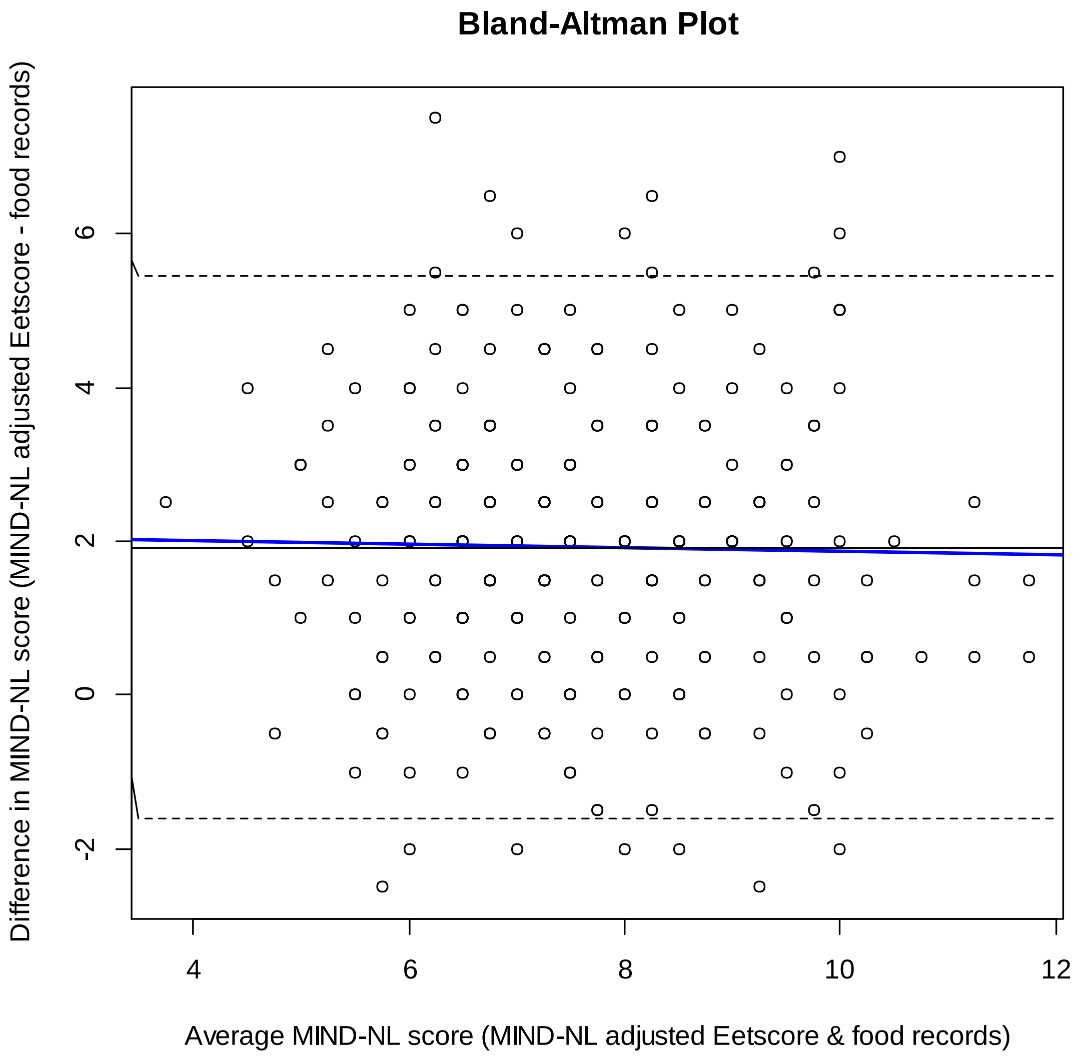


**Supplementary Figure S1**. Bland-Altman plot of the differences in scoring of the MIND-NL diet with the food record and the MIND-NL-Eetscore FFQ, plotted against mean of both methods. **Only participants included without subjective memory complaints (n=292).** Mean difference (black solid line), 95% limits of agreement (1.96 x SD of mean difference; black dashed line), and linear regression line (blue solid line) are included.

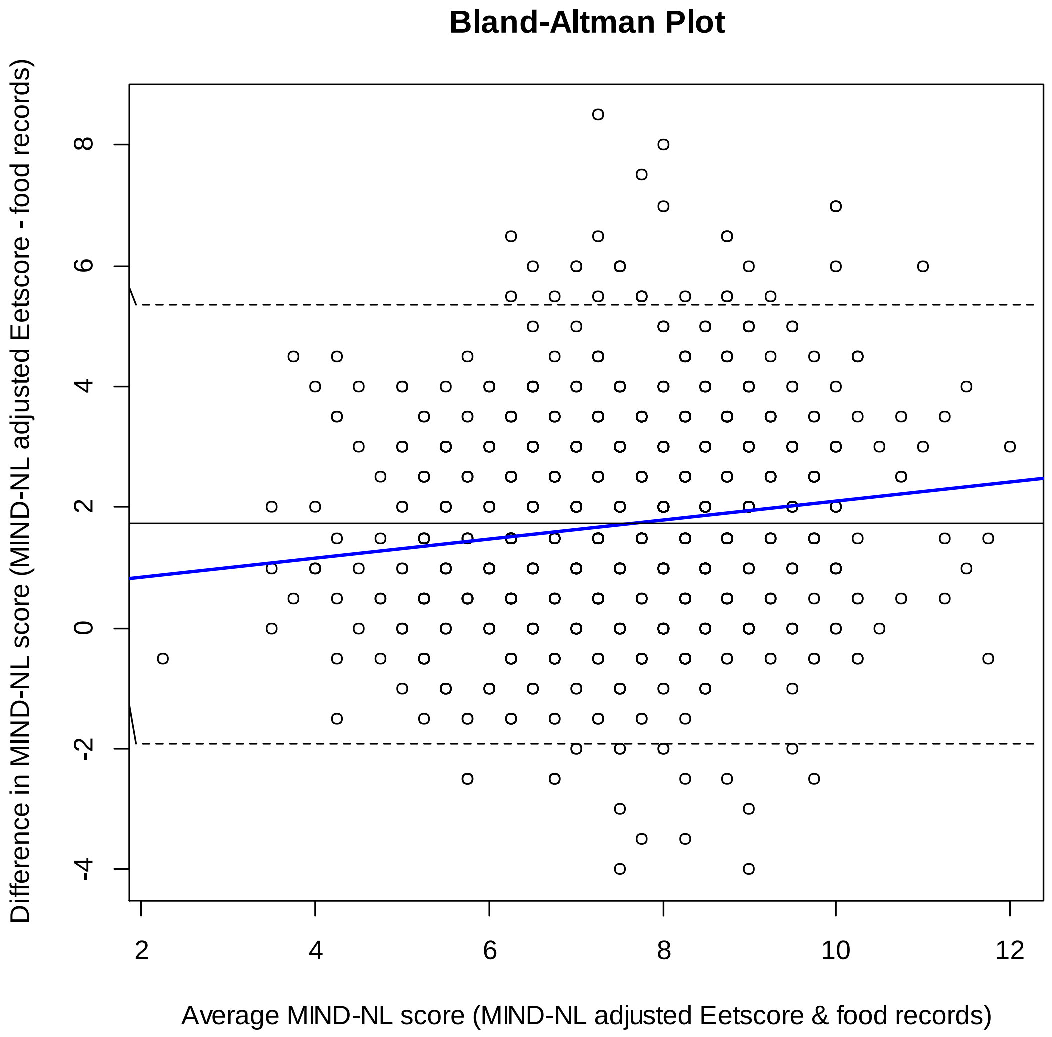


**Supplementary Figure S2**. Bland-Altman plot of the differences in scoring of the MIND-NL diet with the food record and the MIND-NL-Eetscore FFQ, plotted against mean of both methods. **Only participants included with subjective memory compaints (n=785).** Mean difference (black solid line), 95% limits of agreement (1.96 x SD of mean difference; black dashed line), and linear regression line (blue solid line) are included.

**Supplementary Table S1**. In- and exclusion criteria FINGER-NL study

| **Inclusion** | **Exclusion** |
|---|---|
| 60-79 years of age at pre-screening | Self-reported diagnosis of dementia or mild cognitive impairment |
| Adequate fluency in Dutch to understand the informed consent and complete study questionnaires | Cognitive impairment assessed by the Modified Telephonic Interview for Cognitive Status battery (TICSm score <23) |
| Informed consent to all study procedures | Conditions affecting safe and continuous engagement in the intervention (e.g. under treatment for current malignant diseases), major psychiatric disorders (e.g. major depression, psychosis, bipolar disorder), neurological disorders thought to interfere with cognitive function (e.g. Parkinson's disease, multiple sclerosis), symptomatic cardiovascular disease (e.g. stroke, angina pectoris, heart failure, myocardial infarction), re-vascularization within the last three months, severe loss of vision, impaired hearing or communicative ability, severe mobility impairment, other conditions preventing co-operation as judged by the local study nurse or consulted physician at the local study site) |
| Internet access at home | Simultaneous participation in any other intervention trial at time of pre-screening |
| Presence of ≥2 self-reported modifiable risk factors for cognitive decline<br>• Physical activity<br>• Unhealthy diet<br>• Low mental/cognitive activity<br>• High blood pressure<br>• High cholesterol<br>• High Body Mass Index (BMI): defined as ≥25kg/m$^2$ for 60-69y of age, and ≥28kg/m$^2$ for ≥70y of age, based on self-reported height and weight | Participation in FINGER-NL of another household member to prevent contamination |
| Presence of ≥1 self-reported non-modifiable risk factor<br>• First-degree family history<br>• Subjective cognitive decline/memory complaints | |

**Supplementary table S2**. Product classification according to MIND-NL diet components.

| MIND component | Products[1] | 2016 NEVO codes[1,2] |
| --- | --- | --- |
| **Green Leafy vegetables** | Endive, kale, turnip greens, different forms of lettuce, chard, spinach, purslane, water cress, mustard greens, clarion, bok choy, tayer leaf, Rucola | 7, 8, 16, 38, 46, 48, 51, 52, 65, 129, 140, 146, 561, 563, 675, 676, 677, 952, 959, 1398, 1399, 1892, 2316, 2346, 2736, 3086<br><br>*Composite product, therefore weighted to content of only green leafy vegetables:*<br>65, 1531, 647, 1483, 1818, 1919 |
| **Other vegetables** | Aubergine, celery, cauliflower, Chanterelle, mushroom, Chinese cabbage, green cabbage, celeriac, cucumber, turnip, kohlrabi, leek, Rhubarb, red cabbage, Savoy, Pointed white cabbage, brussels sprouts, sweet corn, bean sprouts, tomato, onions, chicory, white cabbage, carrot, sauerkraut, radish, Black radish, garden cress, asparagus, pickle, peas, string beans, green beans, broad beans, Tjap tjoy, Antroewa, Avocado, garlic, fennel, pepper, Broccoli, courgette, beetroot, Salsify, Artichoke, Cassave, Taro, Yam, sweet potato, pumpkin, Okra, Sopropo, parsnip | 11, 13, 14, 15, 18, 20, 21, 22, 24, 25, 26, 27, 28, 29, 30, 37, 40, 41, 42, 43, 44, 53, 54, 55, 57, 59, 60, 61, 63, 64, 67, 68, 69, 70, 71, 72, 73, 74, 124, 125, 126, 127, 130, 131, 132, 133, 134, 135, 136, 138, 139, 141, 142, 143, 144, 145, 472, 538, 557, 562, 650, 674, 689, 800, 830, 849, 850, 851, 920, 922, 951, 953, 954, 957, 958, 960, 961, 962, 963, 964, 966, 967, 972, 1021, 1115, 1118, 1119, 1133, 1147, 1153, 1154, 1157, 1158, 1161, 1187, 1191, 1371, 1397, 1454, 1524, 1811, 1904, 2109, 2110, 2111, 2112, 2113, 2293, 2315, 2331, 2377, 2378, 2402, 2498, 2524, 2708, 2726, 2727, 2728, 2729, 2730, 2731, 2732, 2733, 2737, 2738, 2739, 2742, 2743, 2900, 3069, 3128, 3134, 3135, 3224, 3225, 3226, 3254<br><br>*Composite product, therefore weighted to content of only other vegetables:*<br>473, 757, 759, 853, 1876, 2710, 2948, 3175, 197, 371, 470, 471, 577, 646, 761, 762, 763, 765, 766, 788, 792, 1485, 1486, 1491, 1493, 1496, 1497, 1533, 1595, 1815, 1816, 1817, 1822, 1823, 1824, 1837, 1877, 2317, 2318, 2319, 2320, 2321, 2347, 2612, 2840, 2949, 2950, 1373, 802 |
| **Berries and strawberries** | Strawberries, blueberries, red berries, blackcurrants, huckleberries, raspberries, cranberries, Goji berries, Mulberries | 148, 152, 153, 154, 155, 157, 159, 161, 1117, 1217, 3445, 3448 |
| **Whole grains** | Oatmeal, barley malt, rye flakes, Buckwheat groats, Oat bran, muesli, whole grain bread types, brown rice, Quinoa, bulgur, whole grain pasta, | 211, 213, 225, 236, 242, 243, 246, 249, 655, 1011, 1014, 1017, 1018, 1019, 1253, 1322, 1395, 1459, 1779, 2008, 2157, 2348, 2349, 2350, 2353, 2354, 2357, 2382, 2494, 2703, 2704, 2782, 2786, 2787, 2788, 2794, 2796, 2797, 2798, 2799, 2800, 2804, 2809, 2811, 2821, 3007, 3058, 3154, 3200, 3209, 3210, 3211, 3212, 3213, 3326, 3046, 3055, 3056, 3057, 3078, 3187, 3188, 3251<br><br>*Composite product, therefore weighted to content of whole grains:*<br>288 |

| | | |
|---|---|---|
| **Nuts** | Almonds, cashew, hazelnut, chestnut, brazil nuts, peanuts, walnuts, peanut butter, pecans, pistachio, macadamia, | 198, 199, 200, 201, 203, 204, 205, 206, 207, 455, 541, 876, 1895, 1896, 1935, 2048, 2367, 2844, 2886, 2887, 2345.<br><br>*Composite product, therefore weighted to content of nuts:*<br>2345 |
| **Fish (not fried)** | Eel, herring, bokking, mackerel, sardines, haddock (liver), stockfish, salmon, cod, plaice, whitefish, hailbut, anchovies, tuna, sprat fillet, trout, whiting, sole, tilapia | 112, 113, 114, 116, 347, 349, 350, 353, 355, 356, 357, 602, 603, 604, 703, 813, 817, 819, 820, 822, 918, 919, 1096, 1100, 1255, 1310, 1315, 1585, 1586, 1588, 1589, 1590, 1605, 1606, 1607, 1609, 1610, 1612, 1613, 1614, 1615, 1616, 1617, 1620, 1624, 1625, 2296, 2297, 2298, 2299, 2765, 2947, 3070, 3071, 3137, 3256, 3318, 3319, 3321, 3322<br><br>*Composite product, therefore weighted to content of fish:*<br>1496, 2612, 2949 |
| **Poultry (no skin, not fried)** | Chicken fillet, chicken | 1392, 1635<br><br>*Composite product, therefore weighted to content of poultry:*<br>2317 |
| **Beans and legumes** | Capuchin, brown beans, white beans, lentils, soybeans, chickpeas, split peas, mung beans, kidney beans | 196, 660, 968, 969, 970, 971, 1095, 1369, 2314, 3049, 3073, 3184, 3185,<br><br>*Composite product, therefore weighted to content of beans and legumes:*<br>3207, 197, 765, 766, 1595, 1822, 1823, 2317, 2319, 2321, 1373, 802 |
| **Olive oil** | Olive oil | 601<br><br>*Composite product, therefore weighted to content of olive oil:*<br>2605, 646, 2317, 2319, 2612, 2178 |
| **High fat cheese** | Edammer, Gouda, cheese spread 40+/48+, camembert, brie, roquefort, creamcheese (60+), Saint Paulin/Port Salut, parmesan, Mon Chou, Gruyere, Emmentaler, Cheddar, Bluefort, sheep's cheese, Old Amsterdam, smoked cheese, Rambol, Stilton, raw milk cheese, clove cheese, Gorgonzola, goatcheese (hard), Mascarpone, Turkish white cheese | 511, 513, 515, 516, 556, 593, 714, 715, 716, 718, 719, 721, 722, 724, 725, 726, 728, 804, 882, 883, 928, 1104, 1108, 1109, 1110, 1112, 1113, 1302, 1487, 1725, 1726, 1809, 1939, 2518, 2678, 2756, 2757, 2758, 2759, 2925, 2995, 3044, 3045<br><br>*Composite product, therefore weighted to content of high fat cheese:*<br>2419, 646, 1491, 1493, 1533, 1817, 1824, 2347, 2178 |
| **Butter and stick margarines** | Dairy butter, stick margarine, herb butter | 310, 1530, 2063, 2067, 3052, 3241<br><br>*Composite product, therefore weighted to content of butter and stick margarines:*<br>2561, 2445, 647, 763, 764, 765, 766, 792, 1483, 1485, 1486, 2320, 2840, 1373, 802 |

| | | |
|---|---|---|
| **Red and processed meat** | Different kind of deli meats (e.g. corned beef, ham, luncheon meat, bacon, pate, chicken fillet),<br>beef (liver), pork (liver), lamb, frankfurter, Casselerrib, Filet americain, minced meat, sausage, entrecote, tartare, burger, fricandeau, processed poultry, sour will, steak, chorizo, schnitzel | 319, 324, 328, 333, 334, 335, 336, 338, 340, 566, 567, 568, 638, 639, 640, 641, 642, 643, 699, 782, 783, 784, 785, 810, 908, 1152, 1155, 1162, 1238, 1239, 1326, 1367, 1368, 1375, 1376, 1390, 1391, 1536, 1538, 1539, 1540, 1541, 1542, 1543, 1544, 1545, 1546, 1547, 1548, 1549, 1550, 1551, 1552, 1553, 1554, 1555, 1556, 1557, 1558, 1559, 1560, 1561, 1563, 1564, 1565, 1568, 1569, 1570, 1571, 1572, 1573, 1574, 1575, 1576, 1577, 1578, 1579, 1601, 1641, 1645, 1646, 1647, 1771, 1772, 1773, 1774, 1775, 1776, 1777, 1789, 2300, 2301, 2302, 2303, 2304, 2306, 2313, 2381, 2654, 2768, 2836, 2883, 2898, 2901, 2902, 2903, 2904, 2905, 2906, 2907, 2908, 2909, 2910, 2911, 2912, 2913, 2914, 2915, 2996, 2997, 3001, 3002, 3003, 3035, 3039, 3324<br><br>*Composite product, therefore weighted to content of red and processed meat:*<br>371, 470, 471, 577, 646, 647, 761, 762, 764, 766, 788, 792, 1491, 1497, 1595, 1815, 1816, 1817, 1818, 1919, 1822, 1823, 1824, 1837, 1877, 2317, 2320, 2321, 2347, 2950 |
| **Take out, fried foods, and snacks** | All different kind of chips;<br>Snacks: Sausage roll, frikandel, croquette, bitterball, fried spring roll, fish fingers, chicken nuggets, croissant with cheese, cheese souffle;<br>Fastfood: fried mussels, fries, pizza | *Chips (portion eq. is 43.24 grams):*<br>122, 264, 267, 269, 546, 618, 619, 620, 1505, 1937, 1943, 2147, 2163, 2370, 2529, 2706, 2923, 2924, 2926, 2927, 2928, 2929, 3235, 3253, 1699<br><br>*Snacks (portion eq. is 90.45 grams):*<br>266, 322, 326, 369, 609, 610, 814, 818, 899, 901, 943, 944, 948, 1506, 1584, 1643, 1644, 1680, 2527, 2551, 2553, 2830, 2951, 3072, 3075<br><br>*Fastfood – each product with each own portion eq.:*<br>1583, 1678, 1679, 2108, 2942, 2943, 2944, 2945, 2946 |
| **Sweets and pastries** | Bread toppings: syrup, (chocolate) sprinkles, chocolate spread, honey, jam, sweetened coconut meat, gingerbread spread;<br>Pie and large cookies: cake, different types of pies, candybars, (fruit) doughnut, beignet, brownie, 'Moorkop' , 'Berliner bol' , all different kind of cookies of a bigger size;<br>Small cookies: gingerbread, granola bar, cereal bar, biscuit, rice cake, all kinds of smaller cookies; | *Bread toppings (portion eq. 15.02 grams):*<br>378, 381, 427, 433, 434, 435, 436, 442, 443, 444, 445, 449, 457, 484, 807, 1962, 1963, 1964, 3063, 1311, 2424, 2489, 2531, 2656, 2657, 2930, 3242, 1886, 2885<br><br>*Pie and large cookies (portion eq. 73.06 grams):*<br>232, 250, 254, 253, 255, 256, 257, 259, 468, 474, 486, 487, 489, 525, 526, 528, 570, 634, 697, 833, 835, 837, 845, 854, |

| | | |
|---|---|---|
| | Chocolates: toffee, bonbon, chocolate piece, M&M's, nougat | 855, 980, 1365, 1366, 1470, 1473, 1475, 1476, 1478, 1479, 1480, 1945, 2009, 2010, 2190, 2371, 2391, 2392, 2393, 2395, 2396, 2399, 2400, 2401, 2403, 2413, 2417, 2430, 2431, 2432, 2568, 2570, 2577, 2595, 2622, 2661, 2662, 2698, 2721, 2722, 2776, 2863, 2869, 2870, 2893, 3060, 3237, 3238, 3239<br><br>*Small cookies (portion eq. 63.72 grams):* 431, 432, 461, 524, 621, 717, 727, 929, 1450, 1451, 1508, 2266, 2373, 2374, 2375, 2376, 2380, 2383, 2386, 2530, 2952, 3067 |
| **Wine** | Red wine, white wine, rose wine | 422, 423, 2142, 2610 |

Abbreviations: NEVO, Dutch Food Composition Database

[1]Product list is based on products eaten at baseline of the FINGER-NL study

[2] NEVO (2016). NEVO-online 2016. Versie 2016/5.0. Stichting NEVO (Nederlands Voedingsstoffenbestand), Zeist. Website: Dutch Food Composition Database | RIVM

**Supplementary Table S3**. Adherence to the MIND-NL diet and its components, Kendall's Tau-b correlation coefficients, and Spearman correlation coefficients between the MIND-NL Eetscore-FFQ and the food records in **participants without subjective memory complaints** (n=292).

| | **Food records*** | | **MIND-NL adjusted Eetscore-FFQ*** | | | | | | | |
|---|---|---|---|---|---|---|---|---|---|---|
| | Mean | SD/ % | Mean | SD/ % | Kappa (κ) | 95% CI | $\tau_b$ | 95% CI | ρ | 95% CI |
| MIND-NL score, mean (SD) | 6.6 | 1.7 | 8.6 | 1.7 | 0.29 | 0.19-0.37 | 0.32 | 0.25-0.40 | 0.44 | 0.34-0.53 |
| 1. Green leafy vegetables | | 1.4 | 23.3 | | 0.07 | 0.04-0.10 | 0.19 | 0.11-0.26 | 0.20 | 0.09-0.31 |
| 2. Other vegetables | | 65.1 | 29.5 | | 0.15 | 0.08-0.23 | 0.21 | 0.14-0.28 | 0.23 | 0.12-0.33 |
| 3. Berries and strawberries | | 19.2 | 17.5 | | 0.29 | 0.19-0.37 | 0.33 | 0.26-0.40 | 0.36 | 0.26-0.46 |
| 4. Whole grains | | 42.1 | 75.7 | | 0.22 | 0.15-0.29 | 0.35 | 0.28-0.42 | 0.37 | 0.27-0.47 |
| 5. Nuts | | 26.7 | 33.9 | | 0.33 | 0.24-0.41 | 0.38 | 0.31-0.44 | 0.41 | 0.31-0.51 |
| 6. Fish | | 33.9 | 67.8 | | 0.24 | 0.16-0.33 | 0.31 | 0.24-0.38 | 0.31 | 0.20-0.41 |
| 7. Poultry | | 17.5 | 36.7 | | 0.14 | 0.07-0.21 | 0.20 | 0.12-0.27 | 0.21 | 0.10-0.32 |
| 8. Beans and legumes | | 9.2 | 19.2 | | 0.10 | 0.03-0.19 | 0.16 | 0.08-0.23 | 0.17 | 0.06-0.28 |
| 9. Olive oil | | 0.0 | 11.3 | | -0.01 | -0.05-0.02 | -0.05 | -0.13-0.02 | -0.05 | -0.17-0.06 |
| 10. Full-fat cheese | | 41.4 | 45.9 | | 0.25 | 0.16-0.34 | 0.28 | 0.21-0.35 | 0.31 | 0.20-0.41 |
| 11. Butter and sticky margarines | | 73.3 | 59.6 | | 0.22 | 0.12-0.31 | 0.24 | 0.17-0.31 | 0.26 | 0.15-0.36 |
| 12. Red and processed meat | | 48.3 | 56.5 | | 0.27 | 0.17-0.36 | 0.29 | 0.22-0.36 | 0.31 | 0.20-0.41 |
| 13. Take out, fried foods, and snacks | | 54.8 | 45.2 | | 0.20 | 0.11-0.30 | 0.23 | 0.16-0.30 | 0.25 | 0.14-0.36 |
| 14. Cookies, pastries, and sweets | | 24.7 | 35.6 | | 0.35 | 0.26-0.43 | 0.39 | 0.33-0.46 | 0.43 | 0.33-0.52 |
| 15. Wine | | 72.3 | 77.1 | | 0.57 | 0.45-0.68 | 0.57 | 0.52-0.62 | 0.57 | 0.49-0.64 |

**Supplementary Table S4**. Adherence to the MIND-NL diet and its components, Kendall's Tau-b correlation coefficients, and Spearman correlation coefficients between the MIND-NL Eetscore-FFQ and the food records in **participants with subjective memory complaints** (n=785).

| | **Food records*** | | **MIND-NL adjusted Eetscore-FFQ*** | | | | | | | |
|---|---|---|---|---|---|---|---|---|---|---|
| | Mean | SD/ % | Mean | SD/ % | Kappa (κ) | 95% CI | $\tau_b$ | 95% CI | ρ | 95% CI |
| MIND-NL score, mean (SD) | 6.7 | 1.7 | 8.4 | 1.9 | 0.29 | 0.23-0.35 | 0.33 | 0.29-0.37 | 0.45 | 0.39-0.50 |
| 1. Green leafy vegetables | | 3.6 | | 29.3 | 0.04 | 0.02-0.07 | 0.11 | 0.06-0.15 | 0.12 | 0.05-0.18 |
| 2. Other vegetables | | 67.5 | | 30.3 | 0.16 | 0.11-0.21 | 0.23 | 0.19-0.27 | 0.25 | 0.18-0.31 |
| 3. Berries and strawberries | | 17.6 | | 16.8 | 0.28 | 0.22-0.33 | 0.34 | 0.30-0.38 | 0.37 | 0.31-0.43 |
| 4. Whole grains | | 43.6 | | 70.7 | 0.29 | 0.24-0.35 | 0.39 | 0.35-0.43 | 0.41 | 0.35-0.47 |
| 5. Nuts | | 26.6 | | 36.9 | 0.27 | 0.22-0.32 | 0.34 | 0.30-0.38 | 0.38 | 0.32-0.44 |
| 6. Fish | | 31.8 | | 64.2 | 0.22 | 0.17-0.27 | 0.27 | 0.23-0.31 | 0.27 | 0.20-0.33 |
| 7. Poultry | | 17.3 | | 37.7 | 0.16 | 0.12-0.21 | 0.22 | 0.18-0.27 | 0.24 | 0.17-0.31 |
| 8. Beans and legumes | | 12.5 | | 21.1 | 0.05 | -0.01-0.10 | 0.08 | 0.03-0.12 | 0.08 | 0.01-0.15 |
| 9. Olive oil | | 0.8 | | 12.6 | 0.03 | 0.00-0.06 | 0.09 | 0.04-0.14 | 0.09 | 0.02-0.16 |
| 10. Full-fat cheese | | 43.3 | | 43.1 | 0.38 | 0.32-0.44 | 0.43 | 0.39-0.46 | 0.47 | 0.41-0.52 |
| 11. Butter and sticky margarines | | 74.9 | | 54.0 | 0.24 | 0.18-0.29 | 0.27 | 0.23-0.32 | 0.29 | 0.23-0.36 |
| 12. Red and processed meat | | 46.9 | | 54.6 | 0.27 | 0.22-0.33 | 0.32 | 0.28-0.36 | 0.35 | 0.29-0.41 |
| 13. Take out, fried foods, and snacks | | 52.9 | | 42.7 | 0.21 | 0.16-0.27 | 0.24 | 0.20-0.29 | 0.26 | 0.20-0.33 |
| 14. Cookies, pastries, and sweets | | 24.1 | | 31.6 | 0.30 | 0.24-0.35 | 0.35 | 0.30-0.39 | 0.38 | 0.32-0.44 |
| 15. Wine | | 77.1 | | 77.7 | 0.55 | 0.48-0.62 | 0.55 | 0.52-0.58 | 0.55 | 0.50-0.60 |

**Supplementary Table S5.** Distribution of characteristics, macronutrients and selected micronutrient intakes across sex-specific tertiles of MIND-NL adherence, based on the food record

| | **T1* † N=391** | | **T2*† N=372** | | **T3*† N=315** | | **P for trend‡** |
|---|---|---|---|---|---|---|---|
| | Mean/ median | SD/ IQR | Mean/ median | SD/ IQR | Mean/ median | SD/ IQR | |
| MIND score | 4.9 | 0.9 | 6.8 | 0.5 | 8.6 | 0.9 | **< 0.001** |
| Range | *1.5 - 6.0* | | *6.0 - 7.5* | | *7.5 - 12.0* | | |
| Age (years) | 67.3 | 4.7 | 67.5 | 4.5 | 67.5 | 4.4 | 0.87 |
| BMI | 28.7 | 4.2 | 28.1 | 4.2 | 27.9 | 4.3 | **0.04** |
| MOCA score | 26.6 | 2.1 | 26.6 | 2.1 | 26.9 | 2.1 | 0.18 |
| **Macronutrient intakes** | | | | | | | |
| Energy (kcal/d) | 1690 | 493 | 1621 | 500 | 1614 | 439 | 0.06 |
| Carbohydrates (g/d) | 167 | 55 | 165 | 58 | 161 | 54 | 0.39 |
| Carbohydrates (E%) | 40.9 | 8.0 | 41.8 | 8.5 | 40.7 | 7.7 | 0.15 |
| Mono- and disaccharides (g/d) | 71 | 30 | 71 | 29 | 70 | 26 | 0.79 |
| Fibre (g/d) | 17 | 6 | 19 | 7 | 22 | 8 | **< 0.001** |
| Fibre (E%/d) | 1.9 | 0.5 | 2.3 | 0.6 | 2.7 | 0.7 | **< 0.001** |
| Total fat (g/d) | 70 | 27 | 66 | 28 | 65 | 23 | **0.005** |
| Total fat (E%) | 35.8 | 7.2 | 34.5 | 8.1 | 34.8 | 7.5 | 0.06 |
| Saturated fat (g/d) | 27 | 10 | 24 | 10 | 21 | 8 | **< 0.001** |
| N-3 fatty-acids (g/d), median [IQR] | 1.2 | 0.9 | 1.3 | 1.1 | 1.7 | 1.5 | **< 0.001** |
| Protein (g/d) | 67 | 22 | 68 | 21 | 72 | 21 | **0.002** |
| Protein (E%) | 16.4 | 3.8 | 17.7 | 3.9 | 18.5 | 3.6 | **< 0.001** |
| Plant protein (g/d) | 26 | 10 | 28 | 10 | 31 | 11 | **< 0.001** |
| Animal protein (g/d) | 41 | 17 | 41 | 18 | 41 | 18 | 0.82 |
| Alcohol (g/d), median [IQR] | 8 | 18 | 3 | 12 | 0.2 | 8 | **< 0.001** |
| Alcohol (E%), median [IQR] | 3.0 | 7.1 | 1. | 4.5 | 0.1 | 3.4 | **< 0.001** |
| **Energy-adjusted micronutrient intakes** | | | | | | | |
| Vitamin A RE/2000kcal, median [IQR] | 684 | 550 | 830 | 8.3 | 906 | 790 | **< 0.001** |
| Beta-carotene ug/2000kcal, median [IQR] | 1168 | 1874 | 1741 | 3273 | 2567 | 3677 | **< 0.001** |
| Thiamin mg/2000 kcal | 1.0 | 0.4 | 1.0 | 0.3 | 1.1 | 0.3 | **< 0.001** |
| Niacin mg/2000 kcal | 16 | 6 | 18 | 5 | 19 | 6 | **< 0.001** |
| Vitamin B6 mg/2000kcal | 1 | 0.5 | 2 | 0.4 | 2 | 0.4 | **< 0.001** |
| Folate ug/2000kcal | 230 | 69 | 283 | 119 | 330 | 114 | **< 0.001** |
| Vitamin B12 ug/2000kcal | 4 | 4 | 6 | 9 | 6 | 8 | **0.03** |
| Vitamin C mg/2000kcal, median [IQR] | 76 | 70 | 92 | 88 | 114 | 97 | **< 0.001** |
| Vitamin D ug/2000kcal, median [IQR] | 2 | 2 | 3 | 2 | 3 | 3 | **0.001** |
| Vitamin E mg/2000kcal, median [IQR] | 10 | 5 | 11 | 5 | 13 | 6 | **< 0.001** |
| Vitamin K ug/2000kcal, median [IQR] | 77 | 97 | 98 | 125 | 140 | 186 | **< 0.001** |
| Magnesium mg/2000kcal | 323 | 65 | 375 | 82 | 425 | 81 | **< 0.001** |
| Iron mg/2000kcal | 11 | 2 | 12 | 2 | 13 | 3 | **< 0.001** |
| Calcium mg/2000kcal | 953 | 313 | 1006 | 384 | 1073 | 324 | **< 0.001** |
| Sodium mg/2000kcal | 2395 | 656 | 2388 | 763 | 2308 | 788 | 0.24 |
| Potassium mg/2000kcal | 3119 | 655 | 3554 | 845 | 3858 | 737 | **< 0.001** |
| Zinc mg/2000kcal | 10 | 3 | 11 | 3 | 11 | 2 | **< 0.001** |
| Selenium / 2000kcal, median [IQR] | 42 | 20 | 51 | 23 | 57 | 28 | **< 0.001** |

Abbreviations: BMI, Body Mass Index; MOCA: Montreal Cognitive Assessment

* Standard reporting mean (SD), otherwise stated.

†MIND-NL tertiles are sex-adjusted; *tertiles men* T1:1.5-5.5, T2:6.0-7.0, T3:7.5-12.0, *tertiles women* T1:1.5-6.0, T2:6.5-7.5, T3:8.0-12.0.

‡ Normal distributed data tested with ANOVA, non-normally distributed data tested with Kruskal-Wallis rank sum test for testing differences between MIND diet tertiles.

**Supplementary Table S6.** Absolute average intakes of the MIND-NL components per day, across sex-specific MIND-NL tertiles, based on food record data

| MIND-NL component | T1* N=391 | | T2* N=372 | | T3* N=315 | | P for trend† |
|---|---|---|---|---|---|---|---|
| | Median | IQR | Median | IQR | Median | IQR | |
| 1. Green leafy vegetables (g/d), | 0.0 | 15.0 | 1.7 | 23.3 | 8.3 | 39.9 | **< 0.001** |
| 2. Other vegetables (g/d) | 92.6 | 128.7 | 140.0 | 145.5 | 195.6 | 157.7 | **< 0.001** |
| 3. Berries and strawberries (g/d), | 0.0 | 0.0 | 0.0 | 25.7 | 18.0 | 60.0 | **< 0.001** |
| 4. Whole grains (g/d) | 70.0 | 70.0 | 76.7 | 73.3 | 95.7 | 75.5 | **< 0.001** |
| 5. Nuts (g/d) | 0.0 | 11.7 | 8.3 | 20.4 | 15.0 | 20.0 | **< 0.001** |
| 6. Fish (g/d) | 0.0 | 0.0 | 0.0 | 33.3 | 16.7 | 41.7 | **< 0.001** |
| 7. Poultry (g/d) | 0.0 | 0.0 | 0.0 | 0.0 | 0.0 | 33.3 | **< 0.001** |
| 8. Legumes (g/d) | 0.0 | 0.0 | 0.0 | 0.0 | 0.0 | 18.9 | **< 0.001** |
| 9. Olive oil (g/d) | 0.0 | 0.0 | 0.0 | 1.4 | 0.0 | 3.6 | **< 0.001** |
| 10. High fat cheese (g/d) | 18.3 | 29.9 | 9.1 | 23.3 | 10.0 | 23.3 | **< 0.001** |
| 11. Sticky margarines (g/d) | 2.1 | 8.0 | 1.0 | 4.4 | 0.8 | 3.4 | **< 0.001** |
| 12. Red and processed meat (g/d) | 61.7 | 70.5 | 46.5 | 60.0 | 31.7 | 48.8 | **< 0.001** |
| 13. Fast, fried, and take-out foods (eq./wk) | 2.3 | 4.9 | 0.3 | 2.6 | 0.0 | 0.8 | **< 0.001** |
| 14. Sweets and pastries (eq./wk) | 6.0 | 5.4 | 4.4 | 5.4 | 3.0 | 4.4 | **< 0.001** |
| 15. Wine (ml/d) | 33.3 | 133.3 | 0.0 | 66.7 | 0.0 | 50.0 | **< 0.001** |

Tertiles and absolute food intake are based on food record data.

*MIND-NL tertiles are sex-adjusted; *tertiles men* 1.5-5.5, 6.0-7.0, 7.5-12.0; *tertiles women* 1.5-6.0, 6.5-7.5, 8.0-12.0.

† Kruskal-Wallis rank sum test for testing differences between MIND diet tertiles.

**Supplementary Table S7.** Absolute average intakes of the MIND-NL components per day, across sex-specific MIND-NL tertiles. Absolute intakes based on food record data, sex-specific tertiles based on MIND-NL-Eetscore-FFQ.

| MIND-NL components | T1* N=419 | | T2* N=368 | | T3* N=291 | | P for trend† |
|---|---|---|---|---|---|---|---|
| | Median | IQR | Median | IQR | Median | IQR | |
| 1. Green leafy vegetables (g/d), | 0.0 | 17.4 | 3.4 | 28.6 | 8.3 | 29.1 | **< 0.001** |
| 2. Other vegetables (g/d) | 108.4 | 125.9 | 154.8 | 139.0 | 195.6 | 204.3 | **< 0.001** |
| 3. Berries and strawberries (g/d), | 0.0 | 22.0 | 0.0 | 26.8 | 0.0 | 36.3 | **< 0.001** |
| 4. Whole grains (g/d) | 70.0 | 67.0 | 86.7 | 69.8 | 83.2 | 80.0 | **0.002** |
| 5. Nuts (g/d) | 5.0 | 16.7 | 8.3 | 18.3 | 14.3 | 25.0 | **< 0.001** |
| 6. Fish (g/d) | 0.0 | 8.3 | 0.0 | 33.3 | 0.0 | 40.0 | **< 0.001** |
| 7. Poultry (g/d) | 0.0 | 0.0 | 0.0 | 0.0 | 0.0 | 9.2 | 0.12 |
| 8. Legumes (g/d) | 0.0 | 0.0 | 0.0 | 0.0 | 0.0 | 5.6 | **0.02** |
| 9. Olive oil (g/d) | 0.0 | 0.0 | 0.0 | 3.3 | 0.0 | 3.3 | **< 0.001** |
| 10. High fat cheese (g/d) | 15.5 | 31.8 | 13.3 | 24.7 | 7.0 | 26.0 | **< 0.001** |
| 11. Sticky margarines (g/d) | 2.0 | 6.3 | 1.2 | 4.7 | 0.8 | 4.1 | **0.02** |
| 12. Red and processed meat (g/d) | 50.0 | 63.3 | 47.0 | 58.0 | 36.7 | 63.0 | **0.008** |
| 13. Fast, fried, and take-out foods (eq./wk) | 1.5 | 3.9 | 0.8 | 2.9 | 0.0 | 2.1 | **< 0.001** |
| 14. Sweets and pastries (eq./wk) | 5.5 | 5.6 | 4.7 | 5.8 | 3.3 | 4.9 | **< 0.001** |
| 15. Wine (ml/d) | 0.0 | 100.0 | 0.0 | 83.3 | 0.0 | 66.7 | **0.003** |

Tertiles are based on MIND-NL FFQ data, absolute food intake is extracted from the food records.

*MIND-NL tertiles are sex-adjusted; *tertiles men* 2.0-7.0, 7.5-9.0, 9.5-14.0; *tertiles women* 2.0-8.0, 8.5-

9.0, 9.5-14.0

† Kruskal-Wallis rank sum test for testing differences between MIND diet tertiles.
